# Pharmacological and molecular insights into linalool-rich *Coriandrum sativum* essential oil: Anticonvulsant, analgesic, anti-inflammatory, and antioxidant potential in rodent models

**DOI:** 10.14202/vetworld.2025.2598-2614

**Published:** 2025-09-06

**Authors:** Juan Pedro Rojas-Armas, Jorge Luis Arroyo-Acevedo, Miriam Palomino-Pacheco, José Manuel Ortiz-Sánchez, Hugo Jesús Justil-Guerrero, Jaime Teodocio Martínez-Heredia, María Elena Salazar-Salvatierra, Mariano Gallo Ruelas, Richard Junior Zapata Dongo

**Affiliations:** 1 Laboratory of Pharmacology, Faculty of Medicine, Universidad Nacional Mayor de San Marcos, Lima, Peru; 2 Laboratory of Biochemistry, Faculty of Medicine, Universidad Nacional Mayor de San Marcos, Lima, Peru; 3 Laboratory of Physiology, Faculty of Medicine, Universidad Nacional Mayor de San Marcos, Lima, Peru; 4 Laboratory of Microbiology, Institute for Research in Biological Chemistry, Microbiology and Biotechnology “Marco Antonio Garrido Malo”, Faculty of Pharmacy and Biochemistry, Universidad Nacional Mayor de San Marcos, Lima, Peru; 5 Department of Nutrition, Instituto de Investigacion Nutricional, Lima, Peru; 6 Doctoral Programme in Health Sciences, Faculty of Medicine, Universidad Nacional Mayor de San Marcos, Lima, Peru

**Keywords:** analgesic, anticonvulsant, anti-inflammatory, antioxidant, *Coriandrum sativum*, cytokines, essential oil, linalool, molecular docking

## Abstract

**Background and Aim:**

*Coriandrum sativum* L. (coriander) has long been valued for its culinary and medicinal uses. *C. sativum* essential oil (CsEO), particularly linalool-rich chemotypes, exhibits diverse biological activities; however, integrated evaluations encompassing neurological, inflammatory, and molecular targets remain limited. This study aimed to chemically characterize Peruvian CsEO and assess its anticonvulsant, analgesic, anti-inflammatory, and antioxidant effects, alongside those of pure linalool, while elucidating potential mechanisms through cytokine modulation and molecular docking of cyclooxygenase (COX) enzymes.

**Materials and Methods:**

CsEO was extracted from Peruvian coriander seeds through steam distillation and analyzed using gas chromatography–mass spectrometry (GC-MS). Antioxidant activity was quantified using the 2,2′-azino-bis(3-ethylbenzthiazoline-6-sulfonic acid) assay. Anticonvulsant effects were tested in BALB/c mice using the pentylenetetrazole-induced seizure model, analgesic activity through the acetic acid-induced writhing test, and anti-inflammatory effects in Holtzman rats using the carrageenan-induced paw edema model. Serum interleukin-1β (IL-1β) and interleukin-6 (IL-6) levels were measured by enzyme-linked immunosorbent assay. Molecular docking evaluated linalool’s binding affinity to COX-1 and COX-2 relative to standard non-steroidal anti-inflammatory drugs.

**Results:**

GC-MS identified linalool as the major constituent (59.80%), alongside α-pinene (8.65%), camphor (8.48%), and γ-terpinene (7.09%). CsEO demonstrated potent antioxidant activity (half-maximal inhibitory concentration [IC_50_] = 32.04 μg/mL), exceeding that of linalool alone (IC_50_ = 152.29 μg/mL). Significant anticonvulsant effects occurred at 200 mg/kg for both CsEO and linalool, increasing seizure latency by up to 87.20% and reducing seizure frequency by ~43%. In analgesic assays, linalool (200 mg/kg) achieved a 93.80% writhing reduction, comparable to tramadol, while CsEO showed strong but slightly lower efficacy. CsEO (200 mg/kg) inhibited carrageenan-induced edema by 51.35% at 4 h, reduced IL-1β by 49.8%, and IL-6 by 26.5%, effects comparable to ibuprofen. Docking revealed moderate linalool affinity for COX-1 (−5.70 kcal/mol) and COX-2 (−6.10 kcal/mol), sharing key hydrophobic interactions with reference drugs.

**Conclusion:**

Peruvian CsEO, characterized by a distinctive linalool-rich chemotype, exhibits significant multi-target pharmacological activities, with synergistic contributions from minor constituents enhancing antioxidant and anti-inflammatory effects. Its integrated efficacy profile and favorable safety indicators highlight CsEO as a promising phytotherapeutic candidate for managing seizures, pain, and inflammation. Further studies should explore chronic models, pharmacokinetics, and formulation strategies to optimize clinical applicability.

## INTRODUCTION

Inflammation is an essential physiological defense mechanism that promotes homeostasis and facilitates tissue repair following injury. However, when sustained, it can become detrimental due to excessive production of inflammatory mediators, leading to tissue damage and impaired healing [1]. Acute inflammation is protective, combating infections and supporting repair, whereas chronic inflammation disrupts molecular and cellular functions, contributing to the development of numerous chronic diseases [2]. Conditions such as cancer, cardiovascular disorders, Alzheimer’s disease, Parkinson’s disease, arthritis, diabetes, and obesity rank among the leading global causes of disability and mortality [3]. As a hallmark of aging, inflammation accelerates the progression of age-related disorders [4] and often coexists with functional impairments that diminish quality of life [5]. Although anti-inflammatory drugs are widely used, their long-term application is associated with serious adverse effects, particularly in the gastrointestinal, renal, and cardiovascular systems [6].

Epilepsy, one of the oldest recognized neurological disorders, affects over 50 million people worldwide, with 75% of patients in low-income countries lacking adequate treatment [7]. Despite the availability of more than 20 anticonvulsant drugs, about one-third of patients continue to experience drug-resistant seizures, increasing the risk of premature mortality, injury, psychosocial complications, and reduced quality of life [8]. Furthermore, the toxicity and side effects of current treatments underscore the need for safer, more effective alternatives.

*Coriandrum sativum* L. (coriander), an aromatic herb of the *Apiaceae* family, has a long history of culinary and medicinal applications. *C. sativum* essential oil (CsEO) possesses diverse bioactivities, including hypoglycemic and antioxidant effects in rats [9], cytotoxicity against multiple tumor cell lines [10], fungicidal action against *Candida* spp. [11], and anti-inflammatory as well as anti-colitis effects in rodent models [12]. Linalool, its principal constituent, exhibits a broad pharmacological spectrum, including anxiolytic, antinociceptive, and anticonvulsant effects in mice [13, 14], hepatoprotective properties [15], antihypertensive action [16], protection against ulcerative colitis [17], and airway inflammation [18].

While linalool has been widely studied, the pharmacological potential of the complete CsEO, particularly chemotypes influenced by geographic origin, remains underexplored. Given that compositional variations may influence bioactivity, it is necessary to chemically characterize and experimentally evaluate region-specific CsEO to determine whether its effects parallel or differ from those of linalool alone.

Although CsEO and its major component, linalool, have each been studied for individual pharmacological properties such as antioxidant, anti-inflammatory, or anticonvulsant activity, existing research remains fragmented and geographically biased. Most studies focus on isolated compounds or CsEO from limited geographic origins, neglecting the fact that essential oil chemotypes vary considerably with environmental, agronomic, and post-harvest conditions. These variations can alter biological activity through synergistic or antagonistic interactions among constituents. To date, no study has comprehensively characterized a Peruvian linalool-rich CsEO or systematically compared its biological activities with those of pure linalool across multiple therapeutic domains. Furthermore, while cytokine modulation and molecular docking studies have been separately conducted for plant-derived compounds, no integrated approach has linked *in vivo* pharmacological effects of CsEO and linalool with *in silico* interaction profiles targeting cyclooxygenase (COX) enzymes–key mediators in inflammation and pain pathways. This lack of multi-tiered analysis limits our mechanistic understanding of how CsEO’s complete phytochemical profile contributes to its therapeutic potential, and whether it offers advantages over single-compound administration.

This study aimed to fill these critical knowledge gaps by conducting the first integrated pharmacological and molecular evaluation of a Peruvian linalool-rich CsEO. Specifically, we sought to:

1. Chemically characterize the CsEO composition using gas chromatography-mass spectrometry (GC-MS) to identify both major and minor constituents
2. Compare the pharmacological efficacy of CsEO and pure linalool in validated rodent models of seizures (pentylenetetrazole [PTZ]-induced), inflammatory pain (acetic acid-induced writhing), and acute inflammation (carrageenan-induced paw edema)
3. Quantify systemic inflammatory modulation by measuring serum levels of interleukin-1β (IL-1β) and interleukin-6 (IL-6) following treatment
4. Elucidate molecular mechanisms by performing docking simulations of linalool with COX-1 and COX-2 to evaluate binding affinities and key interactions relative to standard non-steroidal anti-inflammatory drugs (NSAIDs).

By uniting phytochemical profiling, *in vivo* pharmacology, cytokine biomarker analysis, and *in silico* molecular modeling, this research establishes a mechanistic framework linking chemical composition with multi-target bioactivity. Such an integrated approach is expected to provide robust evidence for the potential development of CsEO, alone or in combination with linalool, as a natural, multi-functional therapeutic agent for managing seizures, pain, and inflammation.

## MATERIALS AND METHODS

### Ethical approval

All animal procedures were approved by the Ethics Committee of the Faculty of Pharmacy and Biochemistry, Universidad Nacional Mayor de San Marcos (Approval Certificate No. 010-CE-UDI-FFB-2023). Animal care was conducted in accordance with established guidelines for the care and use of laboratory animals [19]. This study was conducted in accordance with the Animal Research: Reporting *In Vivo* Experiments 2.0 guidelines.

### Study period and location

This study was conducted from April to December 2023 at the Laboratory of Experimental Pharmacology, Faculty of Medicine, Universidad Nacional Mayor de San Marcos, Peru.

### Chemicals and reagents

All products were purchased from Merck KGaA (Darmstadt, Germany). Tramadol (Catalog No. 1672600, ≥98% purirty), ibuprofen (Catalog No. I4883, ≥98% purity), linalool (Lot No. STBJ6366, 97% purity), carrageenan (Catalog No. C1013), PTZ (Catalog No. P6500, ≥99% purity), 2,2′-azino-bis(3-ethylbenzthiazoline-6-sulfonic acid) (ABTS) diammonium salt (Catalog No. A1888), Trolox (6-hydroxy-2,5,7,8-tetramethylchroman-2-carboxylic acid; Catalog No. 648471), and rat IL-1β and IL-6 enzyme-linked immunosorbent assay (ELISA) kits (Catalog Nos. RAB0277 and RAB0311, respectively).

### Plant material and extraction of essential oils

*C. sativum* seeds were collected in Lima, Peru (12°03’36′′S, 77°02’15′′W). A voucher specimen (Certificate No. 98-USM-MHN-2022) was deposited at the Natural History Museum of the National University of San Marcos for taxonomic identification. CsEO was extracted from coriander seeds through steam distillation, using the immiscibility of water and oil for phase separation [20]. A total of 2 kg of seeds yielded 6.4 mL of essential oil. Residual water was removed using anhydrous sodium sulfate. The resulting essential oil was stored at 4°C until further use.

### GC-MS analysis of the essential oil

The constituents of CsEO were identified using GC-MS. An Agilent 7890A gas chromatograph (Agilent, USA) was used to separate the individual volatile compounds. A J&W 122-1545.67659 DB-5 ms capillary column (60 m × 250 μm × 0.25 μm) was used. Helium was used as the carrier gas at a constant flow rate of 1 mL/min. The oven temperature program started at 70°C, increased at 2.5°C/min to 130°C, then at 4°C/min to 200°C, and finally at 20°C/min to 300°C, with each stage held for 3 min. The total run time was 52.5 min. A 3 μL injection was made using a split mode with a split ratio of 20:1. The identification of compounds was based on mass-to-charge ratios (m/z) obtained from the mass spectrometer. An Agilent 5975C mass spectrometer (Agilent) was used to determine the analyte structural and molecular mass information. Spectral acquisition and compound identification were conducted using the Agilent mass selective detector ChemStation software version E.02.02, G1701EA (Agilent), which was integrated with the GC-MS system. Volatile compounds were identified by comparing their mass spectra with entries from the National Institute of Standards and Technology 2008 mass spectral library.

### Antioxidant assay

The ABTS method was performed as previously described by Kuskoski *et al*. [21]. ABTS radicals were generated by mixing 7 mM ABTS with 2.45 mM potassium persulfate and incubating the mixture for 16 h in the dark at 24°C. The resulting ABTS solution was diluted with double-distilled water to achieve an absorbance of 0.70 ± 0.02 at 734 nm. CsEO and linalool were each diluted in a 4:1 methanol–ethanol mixture to final concentrations of 25, 50, 100, and 200 μg/mL. Each 20 μL sample dilution was mixed with 980 μL of ABTS solution, and the absorbance was measured after 7 min. All experiments were performed in triplicate. Trolox (0–10 μg/mL) was used as a reference standard under the same conditions as those used in the present study. The free radical scavenging activity was calculated based on the absorbance values using the following equation:

Inhibition (%) = [(A_0_ – A_1_)/A_0_] × 100

Where A_0_ is the absorbance of the control and A_1_ is the absorbance of the test sample.

The half-maximal inhibitory concentration (IC_50_) was calculated from the concentration–inhibition curve, representing the concentration required to neutralize 50% of ABTS radicals.

### Experimental animals and their housing

A total of 128 male BALB/c albino mice (8 weeks old, 28 ± 2 g) and 72 male Holtzman rats (8 weeks old, 250 ± 5 g) were used in this study. Animals were obtained from the National Institutes of Health vivarium in Lima, Peru. Animals were housed in clean polypropylene cages under controlled environmental conditions: temperature 24°C ± 1°C, relative humidity 55% ± 5%, and a 12-h light/dark cycle. Before the experiment, the animals were acclimatized and provided *ad libitum* access to water and standard rodent pellets.

### Study design

BALB/c mice were divided into eight groups for the anticonvulsant assay and another eight groups for the analgesic assay, while the anti-inflammatory assay involved nine groups of Holtzman rats. Each group comprised eight animals, which were randomly assigned using a list generated in Microsoft Excel 2019 (Microsoft Corporation, USA). The sample size was based on the previous studies by Souto-Maior *et al*. [14], Islam *et al*. [22], and Rege *et al*. [23] using the same models, and *post hoc* analysis. Doses of 50, 100, and 200 mg/kg were selected based on previous pharmacological studies and preliminary toxicity evaluations by Souto-Maior *et al*. [14], Islam *et al*. [22], and Rege *et al*. [23]. The investigator conducting the assessments was blinded to group assignments.

CsEO and linalool were prepared in saline containing 2% Tween 80 before administration. To accommodate differences in body weight between species, two concentrations were prepared in the vehicle: 10 mg/mL for mice and 100 mg/mL for rats. Dosing volumes were calculated individually based on body weight and administered accordingly.

### Anticonvulsant assay

PTZ was administered to 64 BALB/c albino mice, randomly allocated into eight groups (n = 8) to induce seizures.

- Group I: Vehicle (10 mL/kg) - control
- Group II: Diazepam (4 mg/kg)
- Groups III–V: CsEO at 50, 100, and 200 mg/kg, respectively
- Groups VI–VIII: Linalool at 50, 100, and 200 mg/kg, respectively.

All mice received an intraperitoneal injection of PTZ (60 mg/kg) and were observed for 20–30 min post-treatment. The latency to the first seizure (time from PTZ injection to convulsion onset) and the total number of seizures were recorded [14].

### Analgesic assay

The acetic acid-induced writhing test was performed as described previously by Islam *et al*. [22]. The BALB/c mice were divided into eight groups (n = 8 per group). Writhing was induced through intraperitoneal injection of 1% acetic acid (10 mL/kg) 30 min after treatment.

- Group I: Vehicle (10 mL/kg) - control
- Group II: Tramadol (20 mg/kg)
- Groups III–V: CsEO at 50, 100, and 200 mg/kg, respectively
- Groups VI–VIII: Linalool at 50, 100, and 200 mg/kg, respectively.

Nociceptive responses were defined as characteristic writhing behaviors, including abdominal constrictions and hindlimb extension. The number of writhing responses was recorded for 15 min following acetic acid injection. Writhing inhibition (%) was calculated using the following formula:

Inhibition (%) = [(Mean writhes in control–Mean writhes in test)/Mean writhes in control] × 100

### Anti-inflammatory assay

The carrageenan-induced paw edema model in Holtzman rats was used to evaluate anti-inflammatory activity [23]. Seventy-two rats were randomly allocated into nine groups (n = 8 per group) as follows:

- Group I: Vehicle (saline + 2% Tween 80) - normal control
- Group II: Vehicle + carrageenan - negative control
- Group III: Ibuprofen (100 mg/kg) + carrageenan (positive control)
- Group IV: CsEO (50 mg/kg) + carrageenan
- Group V: CsEO (100 mg/kg) + carrageenan
- Group VI: CsEO (200 mg/kg) + carrageenan
- Group VII: linalool (50 mg/kg) + carrageenan
- Group VIII: linalool (100 mg/kg) + carrageenan
- Group IX: linalool (200 mg/kg) + carrageenan.

One hour after treatment, a 0.1 mL injection of 1% carrageenan solution was administered into the right hind paw plantar region in all groups except Group I. Paw thickness was measured using a Vernier caliper at 0, 1, 2, 4, and 6 h to assess edema progression. The paw swelling percentage was calculated using the following equation:

Swelling (%) = [(T – T_0_)/T_0_] × 100

Where, T is the paw thickness at the measured time point, and T_0_ is the initial (baseline) paw thickness.

The percentage of edema inhibition was calculated as follows:

Inhibition (%) = [1 (% swelling in treated group/% swelling in negative control group)] × 100.

Animals were anesthetized with ethyl ether at the conclusion of the experiment, blood samples were collected through cardiac puncture, and euthanasia was performed using an overdose of sodium pentobarbital.

### Cytokine quantification using ELISA

Serum concentrations of IL-1β and IL-6 were determined using ELISA kits (Merck). The assay was performed using 96-well plates precoated with monoclonal antibodies specific for IL-1β or IL-6. Calibration standards were prepared, and 100 μL of each standard and serum sample was added to the wells, followed by incubation at room temperature for 2.5 h. Plates were washed 4 times with wash buffer, after which detection antibodies were added and incubated for 1 h at room temperature for 1 h. Following a second wash, 100 μL of streptavidin was added and incubated for 45 min. After another washing step, 100 μL of 3,3′,5,5′-Tetramethylbenzidine substrate was added and incubated in the dark at 37°C for 30 min until a blue color appeared. The reaction was terminated by adding 50 μL of stop solution, which turned the solution yellow, and absorbance was measured at 450 nm using a microplate reader (BioTek Instruments, Inc., Vermont, USA). The cytokine concentrations were calculated by interpolating the absorbance values against the standard calibration curve.

### Molecular docking

#### Preparation of the protein and ligand structures

The crystal structures of COX-1 and COX-2 were retrieved from the Research Collaboratory for Structural Bioinformatics Protein Data Bank (PDB) (https://www.rcsb.org/) using the PDB IDs 1EQG and 3NL1, respectively. The bound ligands were removed using the PyMOL academic software (version 2.6.x) (Schrödinger, USA; https://pymol.org/) [24]. Water molecules were removed, hydrogen atoms were added, and the energy-minimized receptor structures were obtained using Swiss-PdbViewer 4.1.0 (Swiss Institute of Bioinformatics, Switzerland; https://spdbv.unil.ch/) [25] and then saved in PDB format.

The three-dimensional conformers of linalool (target compound), ibuprofen, and celecoxib (reference NSAIDs) were downloaded in SDF format from PubChem (https://pubchem.ncbi.nlm.nih.gov/).

#### Docking and analysis of interaction

Linalool and ibuprofen were docked to COX-1 (grid center: X = 28.19, Y = 38.74, Z = 192.63), and linalool and celecoxib to COX-2 (X = 34.93, Y = −28.97, Z = −9.51), using the PyRx virtual screening platform (version 0.8) (The Scripps Research Institute, USA; https://pyrx.sourceforge.io/) [26]. The binding affinities were calculated using AutoDock Vina (version 1.1.2) (The Scripps Research Institute, USA; https://vina.scripps.edu/) and expressed in kcal/mol [27].

Rigid docking was performed, treating both ligands and receptor proteins as rigid bodies. A grid box of 20 × 20 × 20 Å^3^ was defined around the active site of each receptor, centered at the respective coordinates. In AutoDock Vina, the exhaustiveness value for docking was set to 8.

We confirmed the docking validity by calculating the root mean square deviation (RMSD) between the docked poses and their reference crystal structures (1EQG and 3NL1) using PyMOL alignment tools (Schrödinger, USA; https://pymol.org/).

Linalool complexes and reference NSAIDs (ibuprofen for COX-1, celecoxib for COX-2) were analyzed using the Protein–Ligand Interaction Profiler server (https://plip-tool.biotec.tu-dresden.de/plip-web/plip/index) [28] to obtain detailed interaction profiles at the binding sites. Molecular visualizations were generated using the BIOVIA Discovery Studio Visualizer (https://www.3ds.com/products/biovia/discovery-studio/visualization).

### Statistical analysis

Results are presented as mean ± standard deviation and were analyzed using one-way analysis of variance (ANOVA) followed by Tukey’s *post hoc* test for multiple comparisons. Prior to performing ANOVA, the assumptions of normality and homogeneity of variances were verified. The Shapiro–Wilk test was used to assess normality, and Levene’s test was used to evaluate variance homogeneity. All datasets met the required assumptions, allowing for the valid application of ANOVA. All statistical analyses were performed using Statistical Package for the Social Sciences version 21 (IBM Corp., Armonk, NY, USA). A significance threshold of p < 0.05 was applied throughout the study.

All experimental procedures and data analysis protocols are available on request for verification and reproducibility.

## RESULTS

### Phytochemical components

GS-MS analysis identified linalool as the predominant constituent of CsEO, comprising 59.80% of the total composition (Table 1). Other major constituents were α-pinene (8.65%), camphor (8.48%), and γ-terpinene (7.09%), together accounting for 24.22% of the oil. The remaining 12 metabolites represented 15.98% of the composition, indicating a chemotype rich in monoterpenes.

**Table 1:** Chemical composition of CsEO determined by GC-MS analysis.

Peak	Compound name (NIST 08.L)	Rt (min)	RA%	Chemical structure
1	α-pinene	10.60	8.65	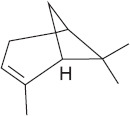
2	Camphene	11.30	1.34	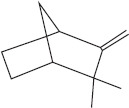
3	β-phellandrene	12.10	0.37	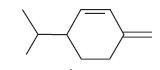
4	β-pinene	12.40	0.61	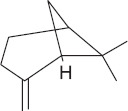
5	β-myrcene	12.50	0.87	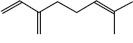
6	Benzene, 1-methyl-2-(1-methylethyl)-	14.20	3.90	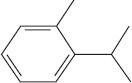
7	D-limonene	14.50	2.90	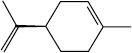
8	γ-terpinene	15.80	7.09	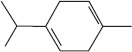
9	Terpinolene	17.10	1.17	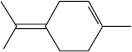
10	Linalool	17.80	59.80*	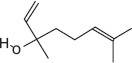
11	Camphor	20.50	8.48	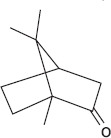
12	Borneol	21.70	0.25	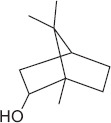
13	Terpineol	22.80	0.39	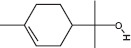
14	Geraniol	25.60	0.86	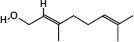
15	Myrtenyl acetate	30.10	0.24	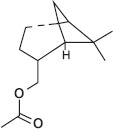
16	Geranyl acetate	32.80	3.09	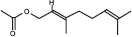

Rt = Retention time, RA = Relative abundance (%), CsEO = *Coriandrum* *sativum* essential oil, GC-MS = Gas chromatography-mass spectrometry, NIST = National Institute of Standards and Technology,

* Bold value indicates the major constituent of the essential oil

### Antioxidant activity

CsEO exhibited strong antioxidant activity, with a potency 4.75-fold higher than that of its principal component, linalool. The IC_50_ value for CsEO was 32.04 ± 0.19 μg/mL, significantly lower (p < 0.001) than that of linalool (152.29 ± 2.22 μg/mL), indicating greater free radical scavenging capacity. Both were less active than the Trolox standard (IC_50_ = 7.69 ± 0.02 μg/mL), which was 4.20-fold and 19.80-fold more potent than CsEO and linalool, respectively (Table 2). These results suggest that the antioxidant activity of CsEO cannot be solely attributed to linalool but may arise from synergistic effects with its minor constituents.

**Table 2:** Antioxidant activity (IC_50_) of CsEO, linalool, and Trolox in the ABTS radical scavenging assay.

Sample	IC_50_ (µg/mL)	Inhibition (%) at 100 µg/mL
CsEO	32.04 ± 0.19	75.7 ± 0.30
Linalool	152.29 ± 2.22	39.6 ± 1.10
Trolox	7.69 ± 0.02	92.9 ± 0.10

IC_50_ = Half-maximal inhibitory concentration, CsEO = *Coriandrum* *sativum* essential oil, ABTS = 2,2′-azino-bis (3-ethylbenzthiazoline-6-sulfonic acid)

### Anticonvulsant activity

**Figure 1:**
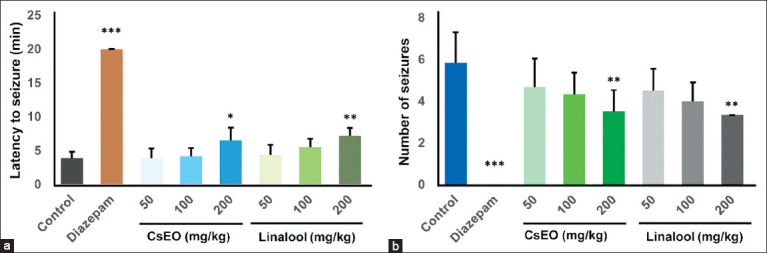
Effect of *Coriandrum sativum* essential oil and linalool on pentylenetetrazol-induced seizures in BALB/c mice (n = 8/group). (a) Latency period for the onset of pentylenetetrazol-induced seizures. (b) Number of seizures during the observation period. Values are expressed as mean ± standard deviation. One-way analysis of variance followed by the Tukey *post hoc* test. *p < 0.05, **p < 0.01 and ***p < 0.001 versus the control group.

Significant anticonvulsant effects were observed only at the 200 mg/kg dose for both CsEO and linalool. Compared with the control group (3.83 ± 0.98 min), CsEO extended the latency to the first seizure to 6.50 ± 1.87 min (p < 0.05; 69.7% increase), while linalool produced an even greater delay of 7.17 ± 1.17 min (p < 0.01; 87.20% increase) (Figure 1a). Both treatments at 200 mg/kg also reduced seizure frequency over 20 min: CsEO (3.50 ± 1.05 seizures; 40.0% reduction) and linalool (3.33 ± 0.82 seizures; 42.9% reduction) compared with the control (5.83 ± 1.47 seizures; p < 0.01) (Figure 1b). No seizures occurred in the diazepam-treated group (4 mg/kg).

### Analgesic activity

**Figure 2:**
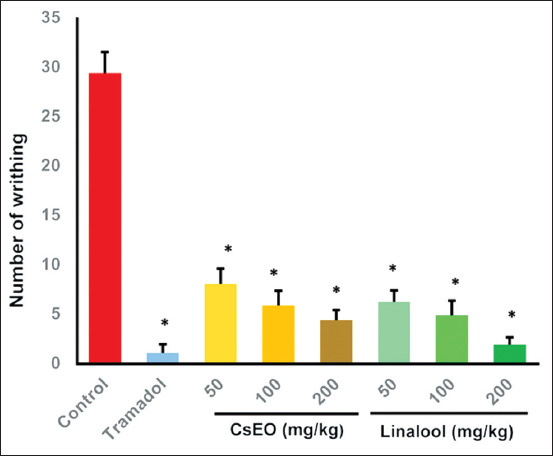
Effect of *Coriandrum sativum* essential oil and linalool on acetic acid-induced writhing in BALB/c mice (n = 8/group). Values are expressed as mean ± standard deviation. One-way analysis of variance followed by the Tukey *post hoc* test. *p < 0.001 versus the control group.

In the acetic acid-induced writhing test, CsEO and linalool both showed significant, dose-dependent analgesic effects. Linalool at 200 mg/kg achieved the greatest inhibition, reducing writhes by 93.80% (1.83 ± 0.75 vs. 29.33 ± 2.16 in the control; p < 0.001), comparable to tramadol (96.6% reduction; 1.00 ± 0.89 writhes; p > 0.05). All tested doses (50, 100, and 200 mg/kg) of both treatments significantly reduced writhing compared with the control (p < 0.05) (Figure 2). At equivalent doses, CsEO was less potent than linalool.

### Anti-inflammatory activity

**Figure 3:**
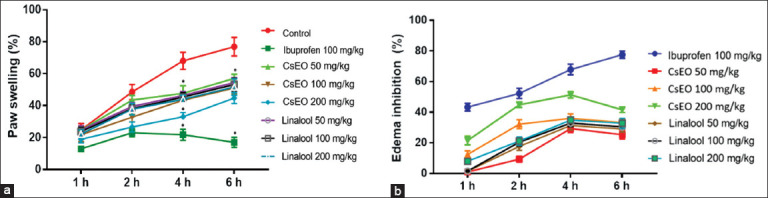
Effect of *Coriandrum sativum* essential oil and linalool on carrageenan-induced paw edema in Holtzman rats (n = 8/group). (a) Paw swelling (%) and (b) inhibition of rat paw edema. Values expressed as mean ± standard deviation. One-way analysis of variance followed by Tukey’s *post hoc* test. *p < 0.001 versus the negative control group (vehicle + carrageenan).

In the carrageenan-induced paw edema model, both CsEO and linalool produced significant, dose-dependent anti-inflammatory effects. At 4 h, CsEO at 200 mg/kg inhibited edema by 51.35% ± 2.08%, compared with 67.91% ± 3.43% for ibuprofen (p < 0.001), while linalool achieved 34.89% ± 1.91% inhibition. Significant effects emerged from the second hour post-treatment (Figure 3a). At 6 h, ibuprofen achieved the highest inhibition (77.61% ± 2.35%), exceeding both CsEO and linalool (Figure 3b).

**Figure 4:**
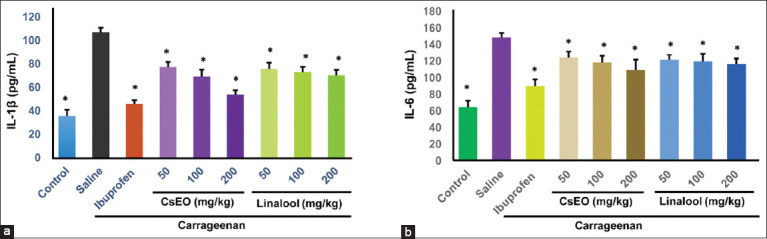
Effect of *Coriandrum sativum* essential oil and linalool on serum levels of interleukin-1β (IL-1β) and interleukin-6 (IL-6) in carrageenan-treated Holtzman rats (n = 8/group). (a) Represents IL-1β levels and (b) IL-6 levels. Values are expressed as mean ± standard deviation. One-way analysis of variance followed by Tukey’s *post hoc* test. *p < 0.001 versus the negative control group.

Cytokine analysis revealed that CsEO (200 mg/kg) reduced IL-1β by 49.80% (53.67 ± 3.80 pg/mL vs. 107.00 ± 3.79 pg/mL control; p < 0.001), comparable to ibuprofen (45.67 ± 3.34 pg/mL; p > 0.05). The same treatment decreased IL-6 by 26.5% (109.33 ± 12.36 pg/mL vs. 148.67 ± 5.24 pg/mL control; p < 0.001) (Figure 4). Linalool also lowered cytokine levels but was less effective than CsEO.

### Molecular docking

**Figure 5:**
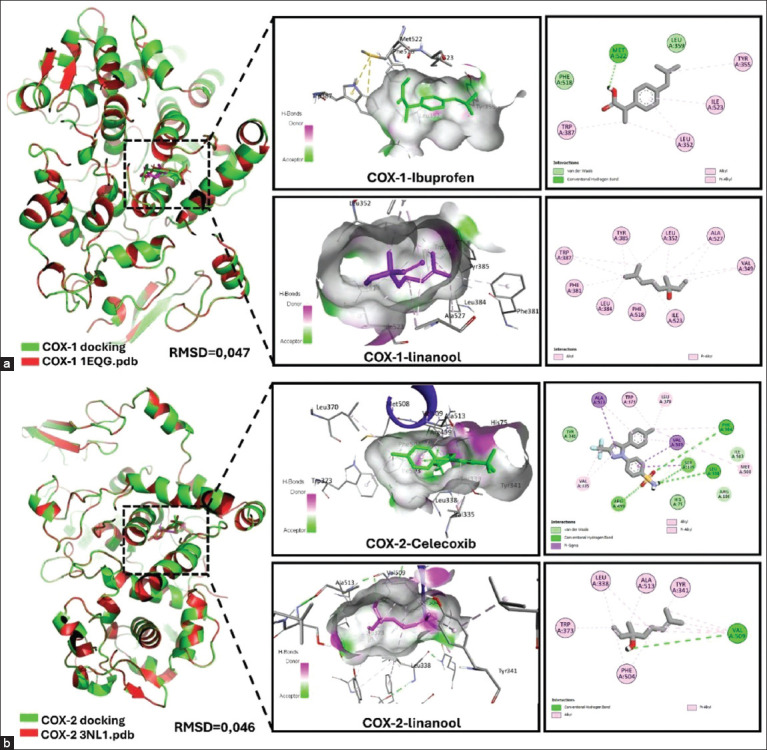
Molecular docking of linalool with cyclooxygenase (COX)-1 and COX-2: ligand binding sites and interaction profiles. (a) COX-1 interacts with linalool and ibuprofen ligand binding sites and interaction profiles. (b) COX-2 in complex with linalool and celecoxib.

Docking simulations showed that linalool bound to COX-1 and COX-2 with affinities of −5.70 kcal/mol and −6.10 kcal/mol, respectively, lower than the reference drugs ibuprofen (−7.60 kcal/mol, COX-1) and celecoxib (−12.10 kcal/mol, COX-2). RMSD values <2 Å (0.047 and 0.046 for COX-1 and COX-2) confirmed model reliability. In COX-1, linalool shared hydrophobic contacts with ibuprofen at Leu352, Trp387, Phe518, and Ile523, and partially overlapped with celecoxib at Tyr341, Phe504, Val509, and Ala513 in COX-2 (Table 3). Although forming fewer hydrogen bonds than the reference drugs, linalool exhibited stable binding conformations and relevant hydrophobic interactions (Figures 5a and b), supporting its potential as a moderate COX modulator.

**Table 3:** Molecular docking parameters and linalool interactions with COX-1 and COX-2 enzymes.

Target protein	Ligand	The grid box coordinates	Binding affinity (Autodock Vina) Kcal/mol	Hydrophobic interactions	Hydrogen bonds	Pi-cation interactions and halogen bond formation	Validation structure (PDB)	RMSD measurement
COX-1	Linalool	X: 28.19 Y: 38.74 Z: 192.63	−5.70	349 Val; 352 Leu; 381 Phe; 384 Leu; 385 Tyr; 387 Trp; 518 Phe; 523 Ile; 527 Ala	-	-	1EQG_A	0.047
COX-1	Ibuprofen	X: 28.19 Y: 38.74 Z: 192.63	−7.60	352 Leu; 355 Tyr; 359 Leu; 387 Trp; 518 Phe; 523 Ile	522 Met	-	1EQG_A	0.047
COX-2	Linalool	X: 34.93 Y: −28.97 Z: −9.51	−6.10	338 Leu; 341 Tyr; 373 Trp; 504 Phe; 509 Val; 513 Ala	509 Val	-	3LN1_A	0.046
COX-2	Celecoxib	X: 34.93 Y: −28.97 Z: −9.51	−12.10	335 Val, 341 Tyr, 370 Leu, 373 Trp, 504 Phe, 509 Val, 513 Ala	75 His; 338 Leu; 341 Tyr; 499 Arg; 503 Ile; 504 Phe	106 Arg	3LN1_A	0.046

PDB = Protein data bank, RMSD = Root mean square deviation, COX-1 = Cyclooxygenase-1, COX-2 = Cyclooxygenase-2

## DISCUSSION

### Chemical characterization of peruvian CsEO

To the best of our knowledge, this is the first study to chemically characterize the CsEO cultivated in Peru using GC-MS, revealing a distinctive linalool-rich chemotype (59.80%). This high linalool content is consistent with reports from other countries, such as European samples (58.00%–80.30%) [29], Iran (57.57% and 59.20%) [12, 30], Slovakia (66.07%) [31], Sudan (66.70%) [32], Egypt (40.90%) [9], India (55.42%) [33], and Argentina (81.70%) [34]. –Yet the Peruvian CsEO displays a unique secondary metabolite profile, particularly due to notable proportions of α-pinene (8.65%), camphor (8.48%), and γ-terpinene (7.09%). These compositional features suggest a chemotype shaped by the local agroecological context, including factors such as altitude, climate, and soil conditions, which are known to modulate terpene biosynthesis [35]. Our finding that the Peruvian CsEO exhibited superior antioxidant activity compared to pure linalool underscores the biological relevance of this chemical profile, suggesting a synergistic contribution of its minor constituents. These findings align with prior reports on monoterpenes of CsEO but also introduce novel evidence of enhanced bioactivity due to geographic variation. In contrast to other studies that have focused solely on linalool or CsEO from different origins, our results highlight the unique composition of the Peruvian oil’s potential therapeutic advantage, which may translate into broader or more potent pharmacological effects due to synergistic interactions beyond linalool alone.

### Antioxidant activity and synergistic effects

The Peruvian CsEO demonstrated superior antioxidant activity compared with pure linalool, with an IC_50_ value of 32.04 μg/mL versus 152.29 μg/mL, respectively (Table 2). This finding indicates that the antioxidant potential of CsEO cannot be attributed solely to its major component, but rather suggests a synergistic effect among multiple constituents. Monoterpenes such as α-pinene and γ-terpinene, known for their radical-scavenging properties, likely enhance the total antioxidant capacity [30]. This effect is biologically relevant because oxidative stress, mediated by reactive oxygen species and reactive nitrogen species, plays a key role in the pathogenesis of inflammatory and degenerative diseases by disrupting cell signaling, damaging biomolecules, and promoting mitochondrial dysfunction and cellular senescence [36]. Therefore, the strong antioxidant profile of CsEO could contribute to its anti-inflammatory efficacy, as observed in the carrageenan-induced paw edema model (Figure 3) and the reduction in inflammatory markers (Figure 4). These results are consistent with those of previous studies: Ahmed *et al*. [32] reported 30.1% antioxidant activity in CsEO from Sudan, while Kačániová *et al*. [31] observed 51.05% 2,2-Diphenyl-1-picrylhydrazyl inhibition in samples from Slovakia. However, our data highlight a more potent antioxidant effect, which may reflect the Peruvian oil’s unique chemotypic features. To the best of our knowledge, this is the first report demonstrating such enhanced antioxidant performance of CsEO cultivated in Peru, supporting the idea that geographical origin significantly influences not only the chemical composition but also the functional bioactivity of essential oils. This novel contribution underscores the importance of characterizing region-specific chemotypes when evaluating medicinal plant therapeutic potential.

### Anticonvulsant effects of CsEO and linalool

In this study, CsEO and its major constituent, linalool, exhibited significant anticonvulsant effects at a dose of 200 mg/kg, as demonstrated by increased seizure latency and reduced seizure frequency in the PTZ-induced seizure model (Figure 1). These results reinforce the therapeutic potential of CsEO and linalool in the management of seizures and are consistent with previous findings. Emamghoreishi and Heidari-Hamedani [37] reported the anticonvulsant activity of CsEO from Iran, while Souto-Maior *et al*. [14] showed that 150 mg/kg linalool significantly delayed seizure onset in mice. Similarly, De Sousa *et al*. [38] confirmed the anticonvulsant properties of both linalool enantiomers and racemates at doses of 200–300 mg/kg, further validating their efficacy. Our study not only confirms these effects at a standardized dose using both the whole essential oil and isolated linalool but also provides the first report of such effects for Peruvian CsEO, contributing novel data on a previously uncharacterized chemotype. This is particularly relevant given the potential for bioactivity to be influenced by regional variations in chemical composition.

The anticonvulsant activity of linalool has been linked to multiple neuropharmacological targets. Linalool may act as a competitive antagonist of L-[^3^H]glutamate binding, attenuating N-Methyl-D-Aspartate (NMDA)-induced excitotoxicity [39], as well as a non-competitive inhibitor of [(3)H]MK801 binding at the NMDA receptor [40]. Additionally, linalool is known to block voltage-gated sodium channels, thereby reducing neuronal excitability [41], and to inhibit neuronal nicotinic acetylcholine receptors, which may indirectly modulate gamma-aminobutyric acid type A (GABAA) receptor activity [42]. These multimodal actions may collectively underlie the observed anticonvulsant effect and indicate that linalool is a promising lead compound. Notably, the fact that the whole CsEO matched the efficacy of isolated linalool in seizure protection suggests that other essential oil components may act synergistically or contribute additively to the overall effect, a hypothesis that warrants further investigation. Thus, our findings expand current knowledge by demonstrating that a linalool-rich Peruvian CsEO exerts anticonvulsant effects comparable to those of pure linalool and CsEOs reported from other regions, confirming its relevance as a potential phytotherapeutic agent.

### Analgesic effects in the acetic acid-induced writhing test

CsEO and its main constituent, linalool, exhibited marked analgesic effects in the acetic acid-induced writhing test, a widely accepted peripheral nociception model. Linalool reduced writhing responses by 93.8% at 200 mg/kg, a result nearly equivalent to that of the reference drug tramadol (96.6%) (Figure 2). These findings underscore the efficacy of both the whole essential oil and the isolated compound in modulating inflammatory pain. The results are consistent with previous reports demonstrating the antinociceptive properties of linalool. For instance, Souto-Maior *et al*. [14] showed a significant reduction in nociceptive behavior in mice, and Batista *et al*. [43] reported its effectiveness in alleviating mechanical hypersensitivity in both neuropathic and inflammatory models, suggesting a broad analgesic spectrum.

Mechanistically, linalool exerts its effects through multiple targets: potentiation of GABAA and glycine receptors [44], inhibition of NMDA receptor-mediated glutamatergic transmission [40], blockade of voltage-gated sodium channels [41], and suppression of transient receptor potential ankyrin 1 (TRPA1) and voltage-gated Ca^2+^ channels [45], all contributing to decreased neuronal excitability. In addition, the inhaled form of linalool has been associated with the activation of orexin-1 receptor signaling in the locus coeruleus, a brainstem region critical for descending pain modulation [46]. Although some inhalation studies suggest TRPA1-independent mechanisms [47], the evidence from direct administration supports transient receptor potential channel inhibition as a key pathway [45].

### Comparative analgesic and anti-inflammatory profiles

A novel aspect of our findings lies in the direct comparative evaluation between CsEO and pure linalool at identical doses, showing that the whole oil also produced robust antinociception while linalool elicited a slightly more potent effect. This suggests that minor constituents may synergistically contribute to the analgesic effect. Previous studies have largely focused on isolated compounds, whereas our results emphasize the use of the complete phytocomplex. Given that the writhing test reflects peripheral pain mechanisms sensitive to nonsteroidal anti-inflammatory drugs and opioids, the results position CsEO as a promising natural agent for inflammatory pain management. However, because this model primarily evaluates nociceptive pain mediated by peripheral pathways, the findings cannot be directly extrapolated to neuropathic pain (NP). Further research using central or NP models is needed to assess whether CsEO and linalool retain efficacy in those conditions.

In the carrageenan-induced paw edema model, CsEO and linalool significantly reduced inflammation, with CsEO achieving 51.35% inhibition at 4 h, compared to 34.89% for linalool at the same dose (Figure 3). This dose-dependent effect was more pronounced in the complete essential oil, suggesting the synergistic action of additional constituents with linalool. These findings are consistent with those of previous studies demonstrating the anti-inflammatory potential of CsEO and linalool. Heidari *et al*. [12] reported the anti-inflammatory effects of CsEO in an acetic acid-induced colitis model, while Tekeli *et al*. [17] and Kim *et al*. [18] described the protective effects of linalool in ulcerative colitis and ovalbumin-induced pulmonary inflammation, respectively, involving mitogen-activated protein kinase and nuclear factor kappa B (NF-κB) pathway inhibition. Linalool has been shown to modulate inflammatory responses in psoriasis-like skin inflammation [48], rheumatoid arthritis [49], oxidative stress-related disorders [50], and nephrotoxicity [51], further supporting its broad anti-inflammatory activity. To the best of our knowledge, this is the first report evaluating the *in vivo* anti-inflammatory efficacy of Peruvian CsEO, highlighting its potential as a phytotherapeutic agent and suggesting that regional chemotypic variation may enhance its biological effects beyond those of isolated linalool.

### Cytokine modulation and systemic anti-inflammatory effects

CsEO also reduced systemic inflammation, as evidenced by significant decreases in serum IL-1β (49.8%) and IL-6 (26.5%) levels (Figure 4), outcomes that were comparable to those of ibuprofen. These cytokines play pivotal roles in amplifying the inflammatory response: IL-1β contributes to matrix degradation, oxidative stress, apoptosis, and angiogenesis [52], while IL-6 is a key driver of chronic inflammation and autoimmunity when dysregulated [53]. The observed cytokine suppression suggests that CsEO and linalool may act, at least in part, by modulating upstream signaling pathways, such as NF-κB, as previously reported by Kim *et al*. [18] for linalool. Inhibition of IL-6 has been linked to both anti-inflammatory and antinociceptive effects [54]. While several studies have explored the role of linalool in inflammation, our findings offer novel evidence that a Peruvian linalool-rich CsEO can downregulate systemic proinflammatory cytokines *in vivo*. This supports a broader immunomodulatory mechanism and provides new insight into the therapeutic scope of CsEO in inflammatory diseases.

### Molecular docking and cyclooxygenase interaction

Molecular docking analysis revealed that linalool exhibits moderate binding affinity toward COX-1 (5.70 kcal/mol) and COX-2 (6.10 kcal/mol), values that fall within the reported range (15–5 kcal/mol) considered sufficient for target inhibition [55]. Although these affinities are lower than those of the reference NSAIDs–ibuprofen for COX-1 (7.60 kcal/mol) and celecoxib for COX-2 (12.10 kcal/mol)–the data suggest that linalool may act as a partial modulator of both enzymes. Structural analysis showed that linalool forms hydrophobic interactions with key residues within the active sites: Leu352, Trp387, Phe518, and Ile523 in COX-1 and Tyr341, Phe504, Val509, and Ala513 in COX-2 (Figure 5). These interactions indicate that linalool can stably occupy the catalytic pocket of both isoenzymes despite its weaker binding energy.

Furthermore, RMSD values <2 Å (Table 3) validate the reliability of the docking models. This dual COX binding profile is biologically relevant because it provides a mechanistic explanation for the *in vivo* anti-inflammatory effects. Unlike conventional NSAIDs that often exhibit strong but selective COX inhibition associated with adverse effects, the moderate and potentially non-selective modulation of linalool could offer a more balanced and safer pharmacological profile. This is one of the first studies to evaluate the interaction of linalool with COX isoforms using *in silico* modeling alongside functional anti-inflammatory assays, highlighting its potential as a natural cyclooxygenase modulator. Similar strategies combining docking with structural validation have been applied to evaluate natural COX-2 inhibitors, including flavonoid derivatives and terpenoid compounds from *Zingiber* species, which demonstrated stable binding and favorable interaction energies through docking and MD simulations [56, 57]. These comparative studies reinforce the relevance of linalool’s binding to COX-2, even at moderate affinity, as part of a broader class of natural product-based modulators with therapeutic potential.

### Synergistic interactions and phytochemical complexity

The superior antioxidant, anti-inflammatory, and analgesic effects of CsEO compared to pure linalool at equivalent doses suggest that the biological activity of the oil is not solely dependent on its major constituent. Rather, synergistic interactions between linalool and other monoterpenes such as α-pinene, camphor, and γ-terpinene likely contribute to the observed effects. These compounds have each been reported to possess pharmacological properties that complement linalool’s mechanisms of action [58–65]. For instance, α-pinene has been shown to exhibit anti-inflammatory and antimicrobial activities, camphor demonstrates analgesic and counterirritant effects, and γ-terpinene possesses antioxidant properties. The combined presence of these constituents may enhance CsEO’s efficacy by targeting multiple pathways simultaneously, an effect unattainable by single-compound administration. This underscores the importance of considering whole essential oils as therapeutic agents rather than focusing solely on isolated bioactive components.

### Methodological integration and novelty

This study is, to the best of our knowledge, the first to integrate phytochemical profiling, *in vivo* pharmacological assays, pro-inflammatory cytokine measurement, and *in silico* COX interaction analysis for a Peruvian linalool-rich CsEO. The choice to examine locally sourced CsEO is particularly significant, as essential oil chemotypes can vary based on geographic origin, soil composition, and climatic factors, which may, in turn, affect bioactivity. By characterizing the chemical profile and linking it directly to multiple biological outcomes, this work advances the understanding of CsEO’s therapeutic potential and sets a precedent for region-specific phytopharmacological evaluations. Furthermore, the dual approach, evaluating both the complete essential oil and its principal component, provides a valuable comparative framework for discerning synergistic versus individual compound effects.

### Comparative efficacy and translational potential

The results position CsEO as a promising multi-target natural therapeutic for conditions involving oxidative stress, inflammation, and seizure activity. Its performance in antioxidant and anti-inflammatory assays, coupled with its ability to modulate key cytokines and interact with COX isoforms, supports a pharmacological profile that could be beneficial in the management of complex disorders such as epilepsy with inflammatory comorbidities, chronic pain syndromes, and neurodegenerative diseases. Importantly, the moderate COX-binding affinity observed for linalool suggests that CsEO might exert anti-inflammatory effects with a lower risk of adverse effects commonly associated with potent COX inhibitors. This balanced pharmacodynamic profile increases its translational potential, particularly in populations where long-term NSAID use is contraindicated.

### Limitations and future directions

While the present findings are compelling, certain limitations should be addressed in future research. First, the study utilized acute inflammation and seizure models; chronic models would be necessary to determine sustained efficacy and safety. Second, although cytokine modulation was assessed, broader immunological profiling, including anti-inflammatory cytokines such as IL-10, would help clarify the immunomodulatory spectrum of CsEO. Third, while molecular docking provides valuable mechanistic insights, complementary molecular dynamics simulations and *in vitro* enzymatic inhibition assays could confirm and refine the predicted COX interactions. Finally, given the chemotypic variability of essential oils, comparative studies across different geographic origins would determine whether the observed bioactivity is unique to the Peruvian chemotype or generalizable to other linalool-rich variants.

## CONCLUSION

This study provides the first comprehensive chemical and pharmacological characterization of CsEO cultivated in Peru, revealing a distinctive linalool-rich chemotype (59.80%) with notable proportions of α-pinene, camphor, and γ-terpinene. These compositional features, shaped by the unique agroecological conditions of Peru, contributed to superior antioxidant activity (IC_50_ = 32.04 μg/mL) compared to pure linalool (IC_50_ = 152.29 μg/mL), significant anti-inflammatory efficacy in the carrageenan-induced paw edema model, marked reduction of serum IL-1β (49.8%) and IL-6 (26.5%), as well as potent anticonvulsant and analgesic effects at 200 mg/kg. The multi-target activity profile of CsEO, spanning oxidative stress reduction, inflammatory modulation, seizure suppression, and analgesia, positions it as a promising natural therapeutic for conditions with overlapping pathophysiological mechanisms, such as neuroinflammatory disorders, chronic pain, and epilepsy with inflammatory comorbidities. Its moderate COX-1 and COX-2 binding affinities suggest a potentially safer anti-inflammatory profile compared to conventional NSAIDs, which could be advantageous for long-term use. Given linalool’s established safety in culinary and therapeutic contexts, and the absence of acute toxicity signals in this study, CsEO offers translational potential in both pharmaceutical and nutraceutical applications.

A major strength of this work lies in its integrative methodology, combining *in vivo* pharmacological assays, cytokine profiling, phytochemical analysis via GC-MS, and *in silico* COX docking, to link chemical composition with functional bioactivity. The direct comparison between the whole essential oil and its major component provides novel insight into synergistic phytochemical interactions, emphasizing the therapeutic advantage of the complete phytocomplex over isolated constituents. This study, however, employed single acute experimental models for each biological activity, without validation in chronic or multi-dose regimens. Pharmacokinetic, dose–response, and histopathological assessments were not conducted, and the contribution of individual minor constituents was inferred but not experimentally isolated. Additionally, no a priori power analysis was performed, which may influence the statistical robustness of group comparisons.

Future research should investigate CsEO in chronic disease models, conduct pharmacokinetic and pharmacodynamic studies, and explore advanced delivery systems (e.g., nanoformulations) to enhance bioavailability. Isolating and testing minor constituents, alone and in combination with linalool, would clarify their individual and synergistic contributions. Comparative chemotypic studies across geographic regions could determine whether the observed bioactivity is unique to the Peruvian variant. In summary, the Peruvian linalool-rich CsEO exhibits potent multi-target pharmacological activities that surpass those of pure linalool in several endpoints, likely due to synergistic interactions with its minor constituents. Its balanced efficacy and safety profile highlight its potential as a plant-based therapeutic and support the continued exploration of chemotype-specific essential oils in drug discovery.

## DATA AVAILABILITY

The supplementary data can be made available from the corresponding author upon request.

## AUTHORS’ CONTRIBUTIONS

JPRA and JLAA: Conceptualized the study. JPRA, MPP, JMOS, and RJZD: Conducted the study. MGR and MESS: Statistical analysis. JPRA: Drafted the manuscript. HJJG, JLAA, and JTMH: Validation, investigation, and revised the manuscript. All authors have read and approved the final version of the manuscript.
